# Prevalence, trends, and specialized palliative care utilization in Taiwanese children and young adults with life-limiting conditions between 2008 and 2017: a nationwide population-based study

**DOI:** 10.1186/s13690-024-01315-3

**Published:** 2024-07-03

**Authors:** Shih-Chun Lin, Mei-Chih Huang

**Affiliations:** 1 School of Nursing, Kaohsiung Medical University, No. 100, Shih-Chuan 1st Rd., Sanmin Dist, Kaohsiung, 80708 Taiwan; 2 Center for Long-Term Care Research, Kaohsiung Medical University, Kaohsiung, Taiwan; 3 Department of Medical Research, Kaohsiung Medical University Hospital, Kaohsiung, Taiwan; 4 Department of Nursing, College of Medicine, National Cheng Kung University, No.1, University Road, East Dist., Tainan, 701401 Taiwan; 5 National Tainan Junior College of Nursing, 78, Sec. 2, Minzu Rd., West Central Dist, Tainan, 700007 Taiwan

**Keywords:** Life-limiting condition, Young adult, Pediatrics, Palliative care, Health care utilization

## Abstract

**Background:**

Previous studies have shown a growing need for pediatric palliative care, but there is a lack of knowledge in many countries concerning prevalence of service use among children and young adults with life-limiting conditions. This study aimed to estimate (1) the annual prevalence of children and young adults with a life-limiting condition, and (2) their specialized palliative care and other healthcare utilization.

**Methods:**

Data from the Health and Welfare Data Science Center in Taiwan were used. All children and young adults aged 0–25 years recorded in inpatient or outpatient data, and infants aged < 1 year in death data with a life-limiting condition diagnostic code from 2008 to 2017 were recruited. Poisson regression was used to estimate the crude and adjusted relative risk of prevalence of life-limiting conditions with 95% confidence intervals, adjusted for age and sex, and to evaluate the trend in prevalence of each life-limiting diagnostic groups, in specialized palliative care and other service use.

**Results:**

Data contained 236,250 individuals with a life-limiting condition, of which oncological and congenital abnormalities were the most common. There was an annualized increase over 10 years in the prevalence of life-limiting conditions of 36.4%, from 45,311 cases (59.4 per 10,000 population) to 52,226 cases (81.0 per 10,000 population), with the highest prevalence in individuals aged 21–25 years. All diagnostic groups showed significant increases in prevalence (*p* < .001) with the exception of oncology, circulatory, and “other” group. Specialized palliative care services, including family consultation, shared care, home visits have increased in use over time (*p* < .001), while inpatient hospice has slightly decreased. The highest prevalence of healthcare use was for traditional Chinese medicine (237.1 per 1,000 population in 2017), but this decreased over time (*p* < .001).

**Conclusions:**

Due to a growing trend towards multidisciplinary care, healthcare professionals and policymakers must engage and take action to expand specialized palliative care and integrate delivery of other healthcare services. Traditional Chinese medicine having a decreasing slope, yet still the highest prevalence of use, needs further attention.

**Table:** 

**Text box 1. Contributions to the literature**
• Little is known about how the prevalence of children and young adults with life-limiting conditions changes over time and varies according to diagnosis and age in Taiwan.
• The prevalence of children and young adults with a life-limiting condition in Taiwan has increased markedly between 2008 and 2017.
• The utilization of each specialized palliative care service covered by the national health insurance was less than 6 per 1,000 individuals.
• Public health policies expanding specialized palliative care services, particularly family consultation and shared care, for those with congenital anomalies, are urgently needed.

## Background

Children and young adults with life-limiting conditions have a possibility of early death. Due to improvements in early diagnosis and treatments, survival rates for children and young adults with life-limiting conditions have improved, with an estimated 96 to 116 thousand cases in England as of 2030 [1]. Current research reports a range from 57.5 to 95.7 cases per 10,000 population across countries of those living with a life-limiting condition between 2013 and 2016 [1–4]. The World Health Organization [5] suggests that healthcare for these children and young adults should be integrated with palliative care. Specialized palliative care and other healthcare services need to be coordinated to provide quality care that covers multidisciplinary needs for this population [5]. However, only about 14% of people who need palliative care actually receive it [6]. Understanding how many children and young adults are living with life-limiting conditions and their healthcare use would improve the effectiveness of planning and delivery of services.

A list of the diagnostic life-limiting condition groups was developed through the 10th revision of the International Classification of Diseases (ICD), which was collected from five children’s hospices and the pediatric palliative care services based at the Children’s Hospital in Cardiff, Wales. They categorized life-limiting conditions into eleven categories, including neurology, hematology, oncology, metabolic, respiratory, circulatory, gastrointestinal, genitourinary, congenital, perinatal, and other [1, 7]. Over the years, palliative care demand has been shown to rise among all diagnostic groups in England [1, 7]. However, these studies did not adjust for age and sex. Race and ethnicity have also been found to be a factor in the difference in the prevalence of life-limiting conditions [1, 7]. The number of children and young adults living with life-limiting conditions and receiving specialized palliative care for the world’s largest ethnic group, Han Chinese, is not well understood.

Taiwan has a majority population of people with Han Chinese ancestry [8]. Most Taiwanese adhere to folk religions, Buddhism, Confucianism, or Taoism, or consider themselves nonreligious [9], while death remains a taboo topic [10, 11], similar to China [12]. Ethnicity and religion influence shared norms, values, beliefs, and practices related to illness, suffering, life, and death [11]. For instance, some parents may perceive a child’s illness as stemming from the family’s bad luck [11]. Although progress has been made in reducing gender imbalances and preferences for male children [13], some families still regard the loss of their only male child as the termination of their family’s future generations [14]. As the culture of harmony is highly valued in the parent-health professional relationship, healthcare professionals feel less challenged about discussing palliative care issues when a child is at the end of life [15]. Despite this, Taiwan ranks first in delivering quality palliative care and end-of-life care in Asia [10, 16]. Subsidies for specialized palliative care services have made them affordable to all. Palliative home care services were integrated into the healthcare system through the Taiwan National Health Insurance (NHI) scheme in 1996. Inpatient hospice was introduced in 2000, followed by palliative shared care in 2011 [17, 18]. However, there is insufficient information to support whether the policy interventions are effective in promoting specialized palliative care for Han Chinese children and young adults.

## Methods

The aim of this study is to establish the prevalence of life-limiting conditions and the rates of those receiving specialized palliative care and other healthcare services among children and young adults up to 25 years of each year and to assess their trends in Taiwan.

### Design

#### Data sources

This observational study used administrative data in Taiwan from the Health and Welfare Data Science Center (No. H108169). The NHI was implemented in 1995. The coverage of NHI reached 99.6% of the population, with 93% of hospitals, clinics, and long-term care facilities being included in the NHI system by the end of 2015 [19]. An extract of clinical and demographic information on inpatient, outpatient, and/or emergency care episodes for patient data was recorded, and these data were linked to the Multiple Causes of Death Data if the child had died.

### Case identification

For the estimation of the number and prevalence of life-limiting conditions, an individual child was included in the prevalence calculations if they met the following criteria:

1. They had a diagnosis of one of the life-limiting conditions ICD codes between January 1, 2008, and December 31, 2017,
2. They were ≦ 25 years old,
3. They were residents of Taiwan.

Deceased newborns who had a life-threatening event but without household registration and NHI enrollment were identified by the underlying and additional multiple causes of death recorded from the Multiple Causes of Death Data. The start age recorded at the first hospital episode in each year was used to assign the age category for each individual.

### Life-limiting conditions

The ICD-10 coding framework [1, 7] was used to identify the diagnosis of life-limiting conditions. The coding dictionary was carefully constructed with eleven diagnostic groups although those diagnostic codes related to diseases of the eye and adnexa were not included in this study. The list of ICD-9-CM 2001 version diagnoses and the most likely corresponding ICD-10-CM codes developed by the National Cheng Kung University Research Center for Health Data [20] were used to convert the ICD-10-CM life-limiting diagnoses [1, 7] into the ICD-9-CM codes. The researcher conducted a back conversion by converting the ICD-9-CM codes back to one or more ICD-10-CM codes again to see if each code corresponded to the ICD-10-CM coding dictionary [1, 7]. If there were no equivalent codes, two qualified disease classifiers from a medical center were consulted for the conversion of approximate ICD-9-CM codes. We further identified a cohort of 28,803 children and young adults, with either an ascertained life-limiting condition ICD-10-CM code between January 1, 2016, and December 31, 2016, or with an approximate ICD-9-CM code between January 1, 2015, and December 31, 2015. Five of the approximate ICD-9-CM codes (2598, 7100, 7461, 74,781, 75,555) had sensitivity higher than 50% for receiving a life-limiting condition ICD-10-CM code in 2016, which was 91.6%, 81.8%, 55.6%, 55.7% and 62.8% respectively. The positive predictive values varied widely, from as low as 3.9% for code 7100, 83.3% for code 7461, to as high as 100% for the other three codes. After adding these five approximate codes, except for code 7100 due to low positive predictive value, 89.4% of ICD-10-CM codes [1, 7] had corresponding ICD-9-CM codes.

### Specialized palliative care service and other healthcare utilization

In Taiwan, the NHI scheme’s national specialized palliative care program is based at hospitals and partners with community-based organizations (e.g. home care nursing clinics) to offer coordinated, comprehensive care to Taiwanese residents. When it comes to pediatric patients, specialized palliative care is typically initiated and consulted by hospital-based pediatric primary care teams when the patients’ and their families’ needs are perceived [15]. The specialized palliative care teams are led by at least one physician who has obtained a subspecialty certification in palliative medicine and one nurse who has undergone advanced palliative care training [18].

This national palliative care program covers family consultations (P5407C, 02020B), shared care (P4401B-03B), inpatient hospice (05601 K, 05602 A, 05603B, 05604 K, 05605 A, 05606B), physician visits (05312 C, 05323 C, 05336 C, 05337 C, 05362 C–65 C), and nurse visits for palliative care (05313 C, 05314 C, 05324 C–27 C, 05338 C–41 C, 05366 C–73 C) [18]. These services can be provided continuously in acute care, home, or community care settings, and should not be limited to patients forgoing further disease-directed treatment efforts [15]. Only specialized teams with accreditation can bill for providing the above services; therefore, patients with the above billing codes indicated that they received specialized palliative care. Family consultation codes indicate that a physician with subspecialty certification in palliative medicine has discussed advance care planning directives, goals of care, or documentation of “do not resuscitate” orders with the patient and/or family before a referral to specialized palliative care. Shared care refers to consultations for palliative approach to the assessment and management of physical, psychological, and spiritual symptoms of the patient. Inpatient hospice provides inpatient palliative approach to care in hospitals. Palliative care physician and nurse visits can be provided in homes or long-term care facilities [18].

Other healthcare services not provided by the primary pediatric team, e.g. traditional Chinese medicine and psychotherapy, which are covered by the NHI, were also extracted and analyzed. Both palliative care and other healthcare service utilization records were received from the patients’ inpatient, outpatient, and emergency care orders, Case Type codes, and Gave Kind codes. Children and young adults who received palliative shared care were coded weekly at all hospitals, if the specialized team had visited them at least once a week and at least 30 min per visit [18].

### Data analysis

The prevalence and the 95% confidence intervals (CI) were calculated per 10,000 populations at risk by dividing all children with at least one recorded diagnosis of a life-limiting condition by the population estimates for that mid-year estimates for 2008–2017 according to the Department of Household Registration dataset [21], stratified by age (five-year age groups). Life-limiting diagnoses were categorized into 11 groups based on ICD-9-CM or ICD-10-CM codes. Individuals may be classified into more than one diagnostic group. Poisson regression was used to estimate the crude and adjusted relative risk of prevalence of life-limiting conditions with 95% confidence intervals, adjusted for age and sex, and to evaluate the trend in prevalence of each life-limiting diagnostic groups, in specialized palliative care and other service use. Poisson Regression analyses were performed using the Genmod procedure in the Statistical Analysis Software (SAS).

## Results

**Fig. 1:**
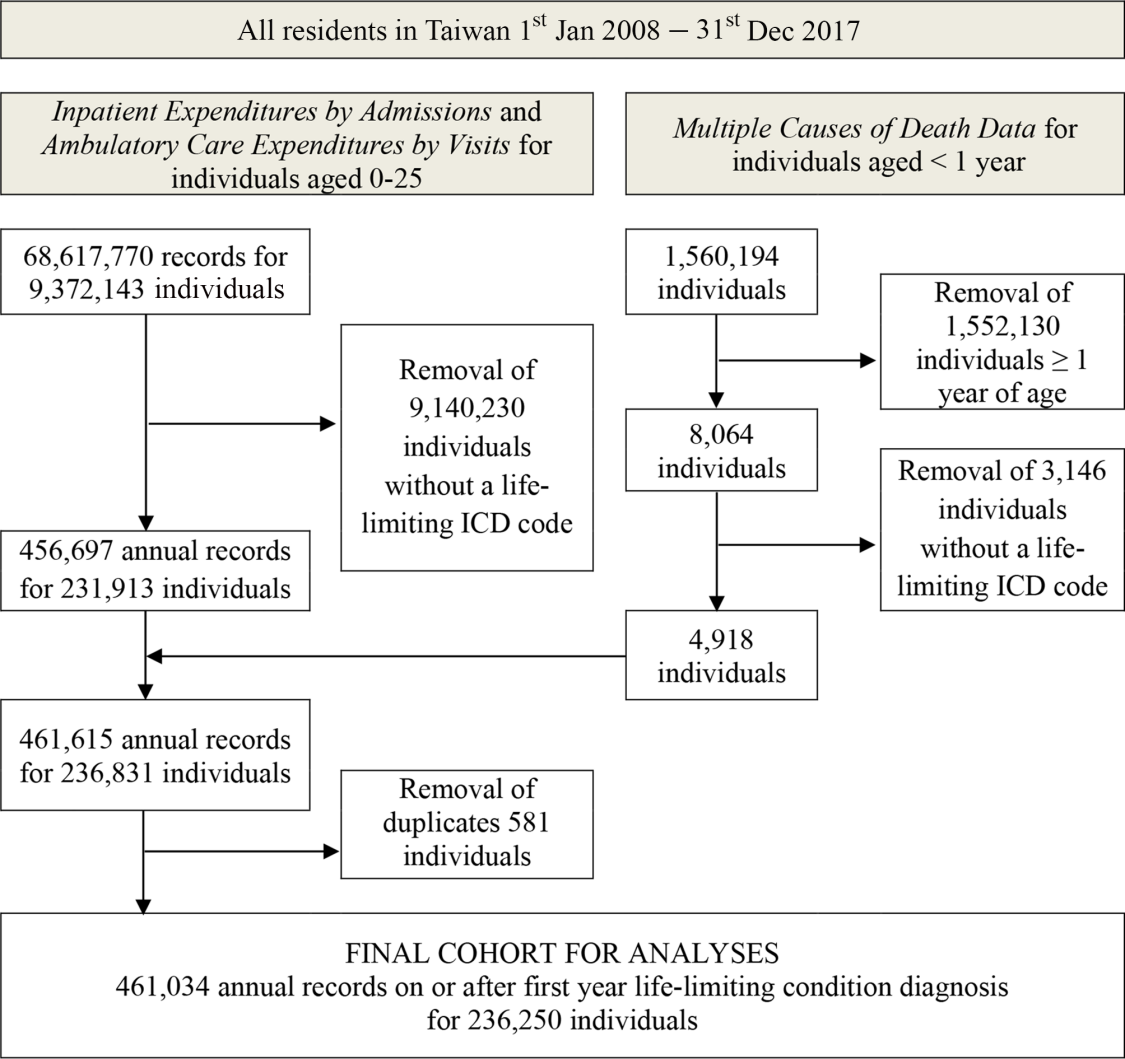
Flow diagram of inclusion criteria

The final analysis dataset contained information on 461,034 annual records for 236,250 individuals (Fig. 1).

### Prevalence

The absolute number and prevalence of children and young adults with a life-limiting condition increased from 45,311 (59.4 per 10,000; 95% CI: 58.8–59.9) in 2008 to 52,226 (81.0 per 10,000; 95% CI: 80.3–81.7) in 2017. Poisson regression showed significant trend for the increases of the annual prevalence of life-limiting conditions after adjusting for sex and age (*p* for trend < 0.001) (Table 1).

**Table 1:** Relative risk of prevalence for children and young adults with life-limiting conditions in Taiwan

Variable	Crude relative risk (95% CI)	Multivariate-adjusted relative risk (95% CI)
**Year**		
2008	1.00	1.00
2009	1.10 (1.09–1.12)***	1.10 (1.09–1.12)***
2010	1.03 (1.02–1.05)***	1.03 (1.02–1.05)***
2011	1.05 (1.03–1.06)***	1.05 (1.03–1.06)***
2012	1.05 (1.03–1.06)***	1.04 (1.03–1.06)***
2013	1.07 (1.06–1.09)***	1.07 (1.05–1.08)***
2014	1.11 (1.10–1.13)***	1.10 (1.09–1.12)***
2015	1.16 (1.15–1.18)***	1.14 (1.13–1.16)***
2016	1.24 (1.22–1.26)***	1.22 (1.20–1.23)***
2017	1.36 (1.35–1.38)***	1.34 (1.32–1.35)***
*P* for trend	< 0.001	< 0.001
**Sex**		
Male	1.00	1.00
Female	0.86 (0.86–0.87)***	0.86 (0.85–0.86)***
**Age**		
< 1 y	1.00	1.00
1–5 y	1.16 (1.14–1.18)***	1.16 (1.14–1.18)***
6–10 y	0.95 (0.93–0.97)***	0.96 (0.94–0.98)***
11–15 y	0.94 (0.93–0.96)***	0.95 (0.93–0.97)***
16–20 y	1.13 (1.11–1.15)***	1.14 (1.11–1.16)***
21–25 y	1.64 (1.61–1.67)***	1.64 (1.61–1.68)***
*P* for trend	< 0.001	< 0.001

***: *p* < .001

### Gender

The prevalence was significantly lower among girls versus boys (relative risk, 0.86; 95% CI: 0.85–0.86, *p* < .001) (Table 1).

### Age

The prevalence of life-limiting conditions in each age group is displayed in Table 2. We observed a significant increase in the relative risk of prevalence with year among all age groups (*p* < .001). The prevalence was significantly lower in the age groups 6–15 years, and significantly higher in both the age groups younger than 5 and 16–25 years (Table 1).

**Table 2:** Prevalence and relative risks across years 2008–2017 for children and young adults with life-limiting conditions in Taiwan

Year	2008	2009	2010	2011	2012	2013	2014	2015	2016	2017	*P* for trend
**No. of patients aged 0–25**											
	45,311	48,701	44,498	44,036	43,388	43,985	44,886	45,655	48,305	52,226	
**Prevalence per 10,000 population**											
**Age 0–25 y**	59.4	65.4	61.2	62.1	62.1	63.7	66.1	68.9	73.5	81.0	
95% Cl	58.8–59.9	64.9–66.0	60.7–61.8	61.5–62.7	61.5–62.7	63.1–64.3	65.5–66.7	68.3–69.6	72.9–74.2	80.3–81.7	
Relative risk	1.00	1.10	1.03	1.05	1.05	1.07	1.11	1.16	1.24	1.36	< 0.001
95% Cl	-	1.09–1.12	1.02–1.05	1.03–1.06	1.03–1.06	1.06–1.09	1.1–1.13	1.15–1.18	1.22–1.26	1.35–1.38	
**Age < 1 y**	54.5	57.5	60.7	60.4	50.4	41.8	54.2	54.4	62.7	69.3	
95% CI	51.2–57.7	54.0-60.9	57.1–64.3	56.7–64.2	47.3–53.5	39.1–44.5	50.9–57.6	51.2–57.6	59.3–66.2	65.6–73.0	
Relative risk	1.00	1.05	1.01	1.11	0.92	0.77	0.99	1.00	1.15	1.27	< 0.001
95% Cl	-	0.96–1.14	0.92–1.10	1.01–1.21	0.85–1.01	0.7–0.84	0.91–1.08	0.92–1.09	1.06–1.24	1.17–1.37	
**Age 1–5 y**	59.9	68.2	69.8	59.7	58.5	60.0	60.1	59.6	78.3	82.1	
95% CI	58.5–61.4	66.6–69.8	68.2–71.4	58.2–61.2	57.0–60.0	58.5–61.6	58.6–61.6	58.1–61.1	76.6–80.0	80.4–83.8	
Relative risk	1.00	1.14	1.00	1.00	0.98	1.00	1.00	0.99	1.31	1.37	< 0.001
95% Cl	-	1.10–1.18	0.96–1.03	0.96–1.03	0.94–1.01	0.97–1.04	0.97–1.04	0.96–1.03	1.26–1.35	1.33–1.42	
**Age 6–10 y**	50.8	58.3	60.7	50.7	47.5	48.4	51.6	53.3	60.0	63.0	
95% CI	49.6–51.9	57.0-59.6	59.4–62.0	49.4–51.9	46.2–48.8	47.1–49.7	50.2–52.9	51.9–54.8	58.4–61.5	61.4–64.5	
Relative risk	1.00	1.15	1.00	1.00	0.94	0.95	1.02	1.04	1.18	1.24	< 0.001
95% Cl	-	1.11–1.19	0.96–1.03	0.96–1.03	0.90–0.97	0.92–0.99	0.98–1.05	1.01–1.08	1.14–1.22	1.20–1.28	
**Age 11–15 y**	45.6	53.3	55.0	50.1	51.0	52.9	52.3	54.9	61.9	65.4	
95% CI	44.6–46.7	52.2–54.4	53.9–56.2	48.9–51.2	49.8–52.1	51.7–54.1	51.0-53.5	53.6–56.2	60.5–63.3	63.9–66.9	
Relative risk	1.00	1.17	1.04	1.10	1.12	1.16	1.15	1.18	1.36	1.43	< 0.001
95% Cl	-	1.13–1.21	1.00-1.07	1.06–1.13	1.08–1.15	1.12–1.2	1.11–1.18	1.14–1.22	1.31–1.4	1.39–1.48	
**Age 16–20 y**	56.9	62.4	62.5	59.4	59.4	61.0	63.3	67.5	68.4	75.8	
95% CI	55.7–58.0	61.1–63.6	61.3–63.7	58.2–60.6	58.2–60.6	59.8–62.2	62.0-64.5	66.2–68.8	67.1–69.7	74.4–77.2	
Relative risk	1.00	1.10	1.05	1.04	1.04	1.07	1.11	1.19	1.20	1.33	< 0.001
95% Cl	-	1.07–1.13	1.02–1.08	1.02–1.08	1.01–1.07	1.04–1.1	1.08–1.14	1.16–1.23	1.17–1.24	1.30–1.37	
**Age 21–25 y**	81.7	85.0	86.9	86.8	89.1	91.4	95.1	98.7	93.9	108.3	
95% CI	80.3–83.0	83.6–86.4	85.5–88.3	85.4–88.3	87.6–90.6	90.0-92.9	93.6–96.6	97.2-100.2	92.4–95.4	106.7- 109.9	
Relative risk	1.00	1.04	1.06	1.06	1.09	1.12	1.16	1.22	1.15	1.33	< 0.001
95% Cl	-	1.02–1.07	1.03–1.08	1.04–1.09	1.07–1.12	1.09–1.15	1.14–1.19	1.19–1.25	1.12–1.18	1.30–1.36	

### Diagnostic group

**Fig. 2:**
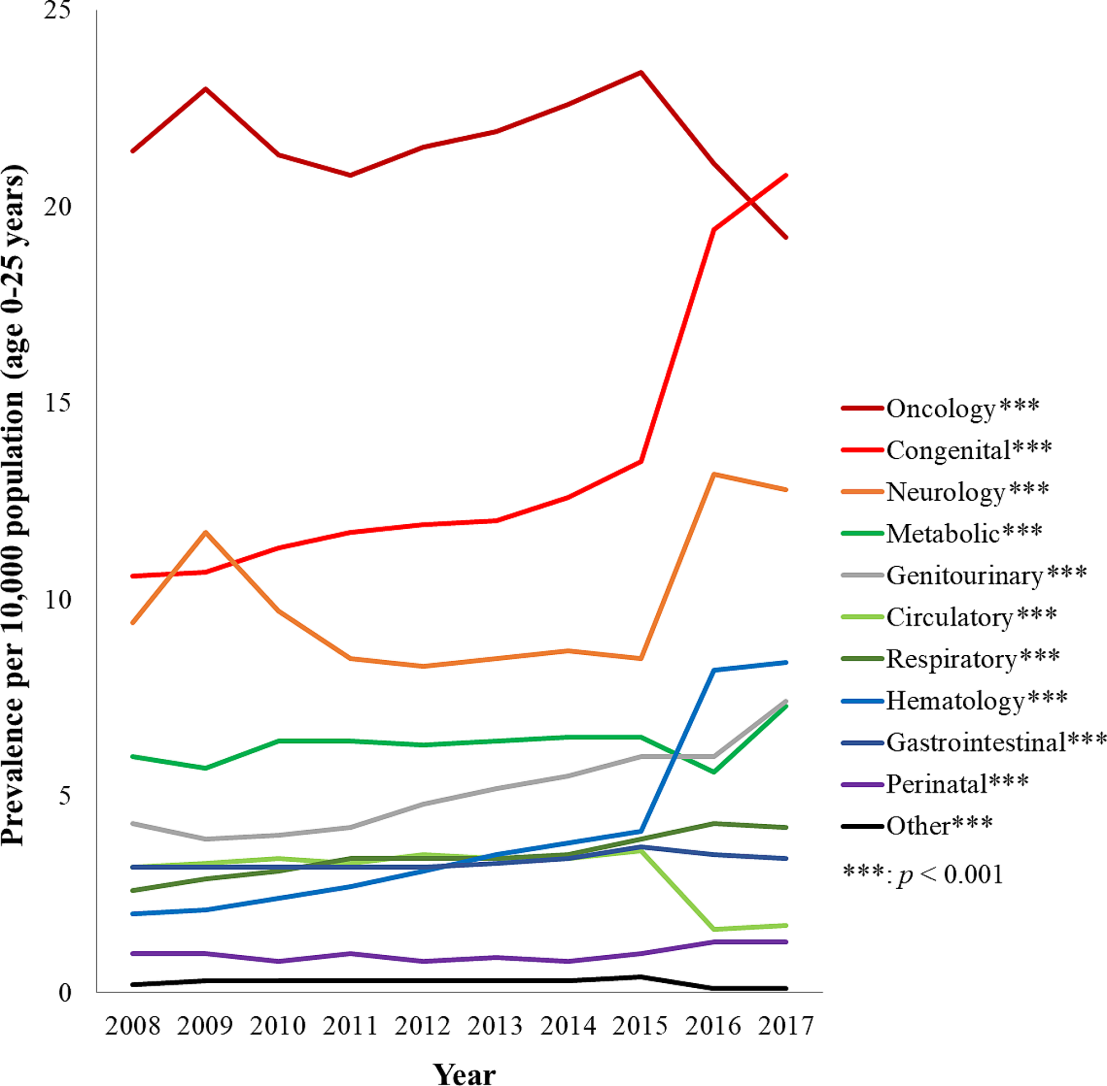
Prevalence of life-limiting conditions in children and young adults in Taiwan for 2008-2017 by diagnostic group

The average annual prevalence of life-limiting conditions was highest for oncology diseases (avg. 21.8 per 10,000), followed by congenital abnormalities (avg. 13.6 per 10,000) over the 10-year time period. The lowest prevalence was “other” (avg. 0.3 per 10,000 population), followed by perinatal (avg. 1.0 per 10,000). All diagnostic groups showed significant increases in prevalence (*p* < .001) with the exception of oncology, circulatory, and “other” group, which had a significant decrease (*p* < .001). Between 2016 and 2017, congenital anomalies rose steeply and have become the highest prevalence, making oncology diagnoses the second most prevalent (Fig. 2).

### Specialized palliative care and other healthcare utilization

**Fig. 3:**
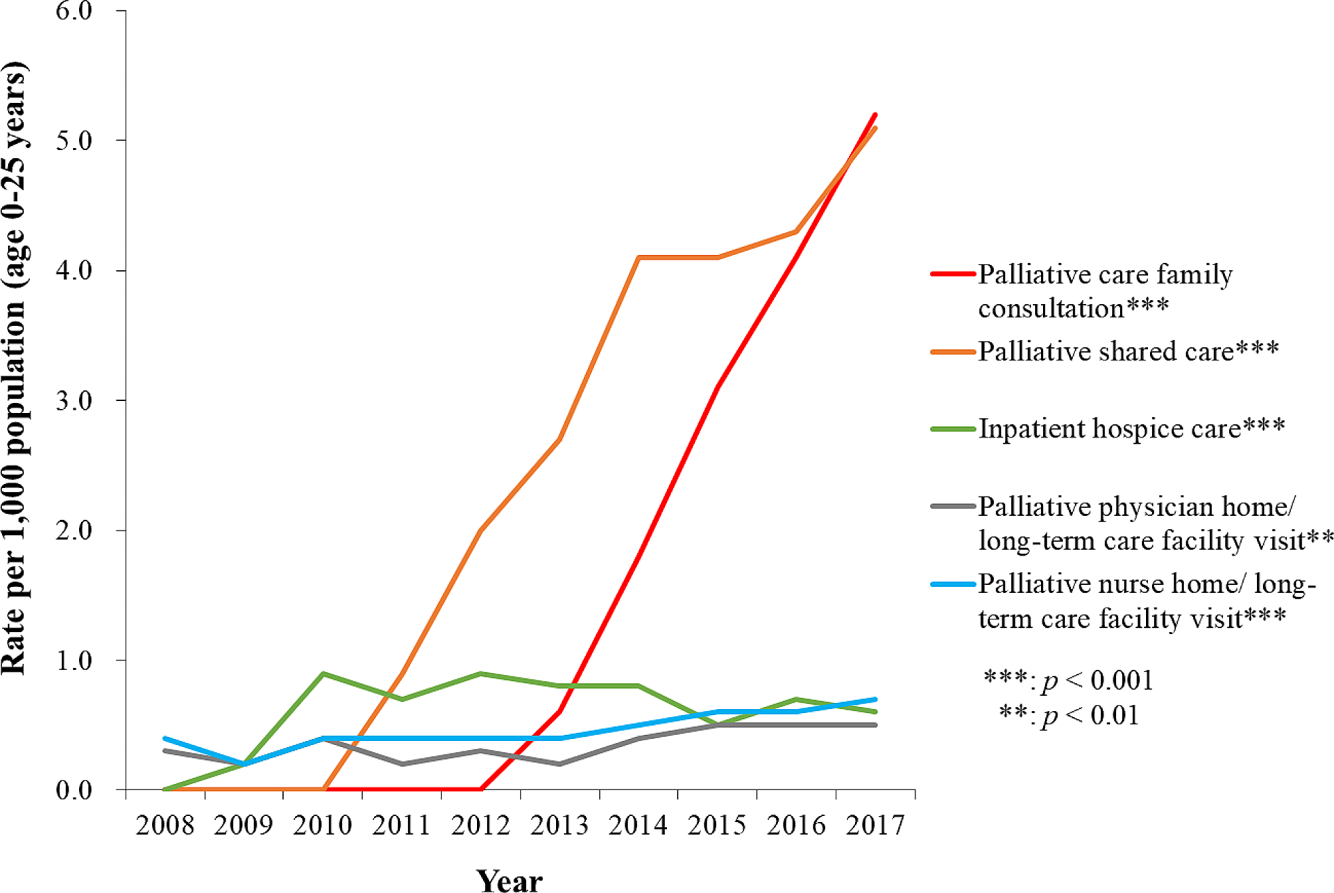
Rate of children and young adults with life-limiting conditions receiving specialized palliative care in Taiwan for 2008-2017

The use of palliative family consultation rose from 0.6 per 1,000 (95% CI: 0.4–0.8) to 5.2 per 1,000 (95% CI: 4.6–5.9) individuals/year since this service was implemented in 2012 (*p* < .001). The use of palliative shared care increased from 0.9 per 1,000 (95% CI: 0.6–1.2) individuals to 5.1 per 1,000 (95% CI: 4.5–5.7) individuals since it was implemented in 2011 (*p* < .001). Figure 3 shows a slight decrease in the rate of inpatient hospice care use in the study time period.

**Fig. 4:**
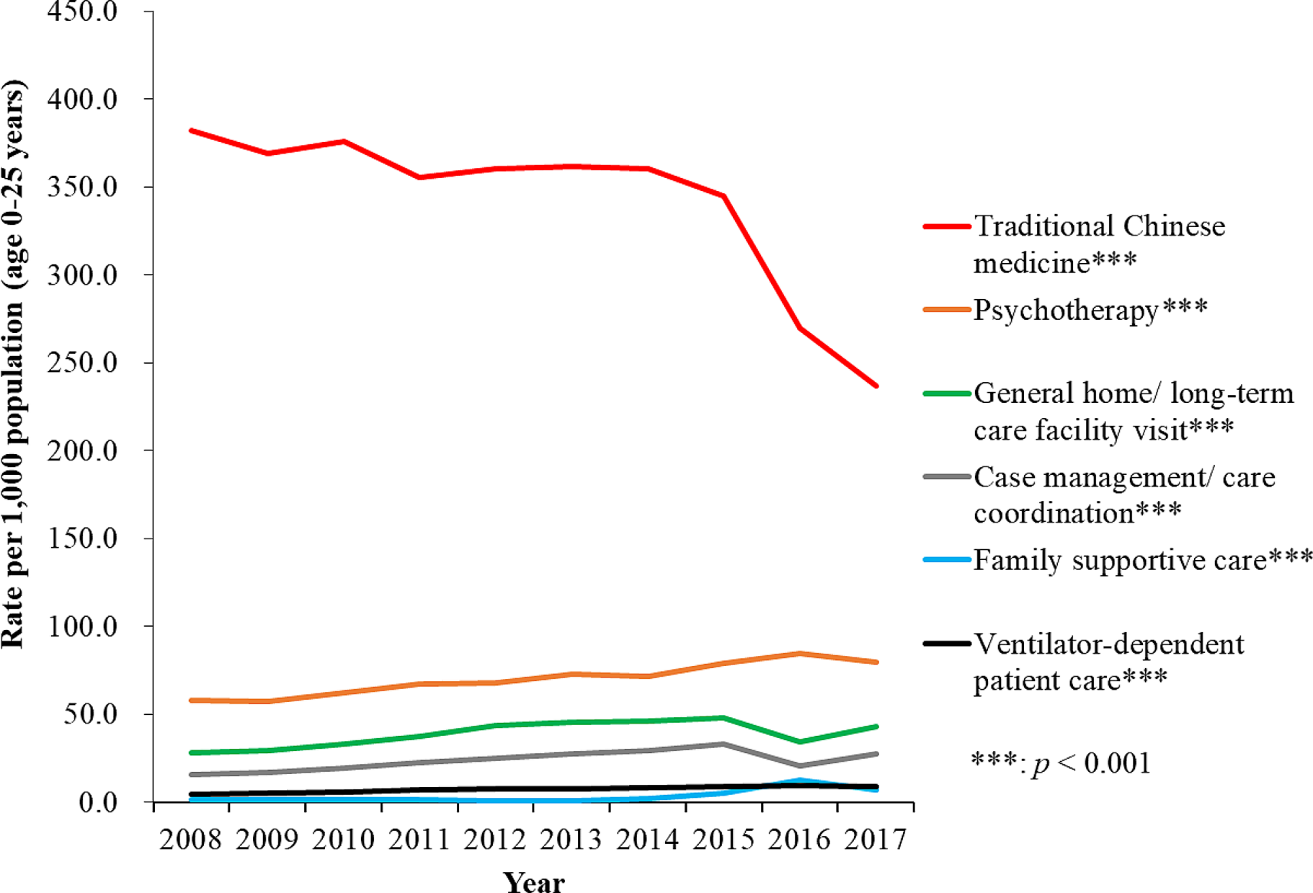
Rate of children and young adults with life-limiting conditions receiving other healthcare services in Taiwan for 2008-2017

As with the other healthcare modalities, the highest rate of use was for traditional Chinese medicine, but this decreased over time from 382.2 per 1,000 (95% CI: 377.7-386.7) individuals in 2008 to 237.1 per 1,000 (95% CI: 233.4-240.7) individuals in 2017 (*p* < .001). The second most highly used services was found in the utilization of psychotherapy, usage rising from 58.1 per 1,000 (95% CI: 56.0-60.3) individuals to 79.7 per 1,000 (95% CI: 77.4–82.0) individuals, a 37.2% increase (*p* < .001), followed by the utilization of general home or long-term care facility visit, usage rising from 28.5 per 1,000 (95% CI: 26.9–30.0) individuals to 42.9 per 1,000 (95% CI: 41.2–44.7) individuals, a 50.5% increase (*p* < .001), and the case management/care coordination, usage increasing from 15.6 per 1,000 (95% CI: 14.5–16.8) individuals to 27.6 per 1,000 (95% CI: 26.2–29.0) individuals, a 76.9% increase (*p* < .001) over the ten-year time period. The rate also increased in the use of family supportive care, from 1.2 per 1,000 (95% CI: 0.9–1.5) individuals in 2008 to 7.3 per 1,000 (95% CI: 6.6-8.0) individuals in 2017, a greater than 6-fold increase (Fig. 4).

### The evidence on deaths

The annual number of deaths among individuals aged 0–25 with life-limiting conditions in Taiwan decreased from 1,491 deaths (32.9 per 1,000 individuals; 95% CI: 31.3–34.6) in 2008 to 1,120 deaths (21.5 per 1,000 individuals with life-limiting conditions; 95% CI: 20.2–22.7) in 2017. Over ten years, an average of 45.5% of deceased children and young adults had a documented life-limiting diagnosis in the year of their deaths. The number of annual deaths among infants under one year of age with life-limiting conditions decreased from 539 deaths (51.1% of all infants with life-limiting conditions) in 2008 to 483 deaths (36.4% of all infants with life-limiting conditions) in 2017. In 2017, 322 infants died within the first twenty-eight days of birth, accounting for 66.7% of the total number of infant deaths, while 208 newborn infants died within their first three days of birth, representing 64.6% of the total number of neonatal deaths.

## Discussion

This study is the first to provide an estimate of number of children and young adults living with life-limiting conditions and receiving specialized palliative care for the world’s largest ethnic group, Han Chinese. The overall prevalence of life-limiting conditions in Taiwan has increased markedly over this ten-year study period, from 59.4 per 10,000 population (95% CI: 58.8–59.9) in 2008 to 81.0 per 10,000 population (95% CI: 80.3–81.7) in 2017. Comparable to previous studies, the prevalence of life-limiting conditions was higher in boys than girls [1, 7]. However, it was observed that girls were more prone to clinical instability than boys [2], such as having a higher risk for urinary tract infection complications [22]. The prevalence of life-limiting conditions in Taiwan was over two times greater than previous estimation for Chinese population in England in 2017 [1]. Different from previous studies [1, 7], the prevalence of life-limiting conditions was highest for oncological disorders in Taiwan, but the annual trend remained relatively static. The exclusion of diagnoses through ICD-10 might lead to a reduction in the reported prevalence of oncological diseases between 2016 and 2017. However, the prevalence of oncology diseases (avg. 21.8 per 10,000 over the ten year) was still higher than that reported in England [1] and other countries with high social-demographic indexes (16.2 per 10,000) [23]. Previous study showed a higher rate of childhood hepatoblastoma in Taiwan (2.67 per million person-y) than the rates in Europe (1.5 per million person-y) and the USA (2.2 per million person-y), which may be related to the genetic background of the Han Chinese ethnicity [24]. As the NHI and the Childhood Cancer Foundation of Republic of China has covered the expenses of cancer treatment, as well as the case management system is increasingly being developed in hospitals, treatment compliance and survival rates for children and young adults with cancer have improved [25, 26]. For instance, the 5-year overall survival rates for children with acute myeloid leukemia without relapse significantly increased from 50.8% during 1996–2007 to 72.5% during 2008–2019 in Taiwan. This may be attributed to the improvement of access to treatments and supportive care significantly decreased death caused by infection [26]. Our study also found a steep decline in use of traditional Chinese medicine, which could be attributed to improved treatment adherence and increased awareness of cancer [27].

Our research has revealed a significant rising trend in the prevalence of congenital anomalies (*p* < .001), making it the most prevalent group since 2017 (20.8 per 10,000 population). This rise may be partly due to the change from using the list of converted ICD-9 codes to the list of diagnostic ICD-10 codes, resulting in an underestimated number of recorded diagnoses between 2008 and 2015. Despite the incidence of newborns with congenital anomalies showing a significant downward trend between 2004 and 2011 [28], congenital malformations remain the leading cause of infant mortality in Taiwan [29]. A previous study reported that less than 30% of Taiwanese children and teenagers with congenital heart disease complied with continual cardiac follow-up care up to their teenage years, potentially leading to avoidable complications and subsequent mortality [30]. With advances in diagnosis, medical technology, surgical interventions, and integrative transitional care, children and young adults were able to live through adulthood [30, 31]. It was predicted that congenital disorders will still be the largest group of life-limiting conditions and will rise to a prevalence of 37.2 per 10,000 population by 2030 [1].

Similarly, as Taiwan’s NHI approved gene therapy for spinal muscular atrophy in children in 2023 [32], future trends to show a rise in the prevalence of neurological diseases may be expected due to an increase in survival rates. During the years of this study, there has been an increase in the prevalence of hematological diseases, which could be attributed to the promotion of multiple screening policies. For instance, Taiwan began a national strategy in 2005 for the elimination of mother-to-child transmission of HIV by offering free testing at antenatal care and medical care services for pregnant women and suspected HIV-infected infants [33]. Since 2017, comprehensive prevention programs and treatment strategies have led to a decrease in HIV transmission and a reduction in the HIV epidemic in Taiwan [34]. With a reduced likelihood of late HIV diagnosis and HIV-related mortality, including suicide and accident mortality [35], this prevalence trend is expected to continue increasing.

Our study found a similar prevalence of genitourinary diseases as compared with that in England [1] and a significant rising trend (*p* = .001). Even though Asian children and adolescents have been reported to have the lowest incidence and prevalence of end-stage renal disease, Taiwan has reported the highest prevalence of individuals with end-stage renal disease undergoing maintenance dialysis globally [36]. Asians having been found to have higher preferences for life-sustaining treatments to prolong life may thus be one the reasons to have a higher prevalence than Western populations [1, 7]. Expanding specialized palliative care service coverage in Taiwan since 2009 led to less use of life-sustaining treatments for elderly patients with end-stage renal disease [37], but the impact on the pediatric population is unclear.

We found the gap between the number of children and young adults with life-limiting conditions and the actual annual number of people receiving specialized palliative care and other healthcare services. Although there was a decline in the annual death rates among children and young adults with life-limiting conditions, which was 21.5 per 1,000 in 2017, it was much lower than the rate of receiving specialized palliative care services. The proportion of patients and families needing specialized palliative care will vary from place to place, depending on the skills and resources available through primary care [38]. Previous studies interviewing Taiwanese pediatric healthcare professionals suggest that refractory pain may be better managed if the child was cared for in an inpatient hospice than by a pediatric primary care team [39]. Although we did find that specialized palliative care services, including shared care, palliative physician and nurse visit, have increased in use over time in Taiwan, inpatient hospice care has a slight decrease since 2012. All children and young adults who receive palliative care services were coded, so it is unlikely that the low percentage of patients is due to a lack of coding for services. For some Taiwanese parents, transferring their child to an inpatient hospice indicated they had given up on their child [11, 40]. A previous study found that two-thirds of Taiwanese pediatric patients who died of cancer received ICU care in their last month of life. This proportion was higher than what was reported in the United States and Korea [41]. Therefore, instead of transferring to inpatient hospices, which were located in an adult care setting, Taiwanese children and young adults may prefer a shared care model where they could be cared for by their primary pediatric care team. This also indicated an increasing need for adequate transition [31] and advance care planning for adolescents and young adults [42], which both currently lack service access in Taiwan.

This study also found that people using general home care service was over 60 times higher rates than those receiving specialized palliative home visits in 2017. One possible reason for this was the fact that specialized palliative care services can only be covered by the NHI if the patients were in at least one of the following life-limiting conditions: advanced cancer, terminal motor neuron disease, senile and presenile organic psychosis, cerebral degeneration, heart failure, chronic airway obstruction, other lung diseases, chronic liver disease and cirrhosis, and renal failure [18]. These conditions are generally adult specific and not relevant to children. After comparing ICD codes of the above diseases [43] with the coding framework of life-limiting conditions [1, 7], we found none of the ICD codes in the hematological, metabolic, circulatory, congenital abnormalities, perinatal, and “other” groups were eligible for specialized palliative care services coverage by Taiwan’s NHI. Therefore, we recommend that policies are needed for expansion of specialized palliative care services to ensure that service access is available to children and young adults in all diagnostic groups.

Despite the observed low rate of palliative home care use, one should be cautious to conclude that children and young adults prefer hospital-based services (shared care or inpatient hospice) over community-based care options [44]. Previous studies suggested that among Taiwanese children and young adults with cancer, specialized palliative care team were involved at a later stage of the disease, with more than one-fifth of them receiving such care within their last 3 days of life [45]. This usually results in not enough time for setting up the outpatient setting, such as adequate equipment available in the home or an access to a palliative care specialized contact. In Taiwan, only persons with specialized training in palliative care can deliver palliative home care, but they may lack pediatric palliative care training. The first documented professional training for pediatric palliative care began in 2014 by the Taiwan Association of Hospice Palliative Nursing, but only included two hours of lecturing in the total 15 days of palliative care training and the practicum [46]. A lack of trust in an adult patient-oriented specialized palliative care team may be one of the reasons for the low rate of use in children and young adults.

This study also found an increased usage of chronic care for ventilator-dependent patients (*p* < .001). This may indicate a growing number of families were home-caring for children and young adults who were dependent on ventilators and other highly technical care. For patients with long-term care needs, with the family having ability to care independently for their child, some suggested that palliative home care provides an important option for respite care [40]. To improve community-based palliative care for this population, policy within the NHI in Taiwan should emphasize home-based multi-disciplinary approaches to support family caregivers, e.g. in-reach emergent technical support or short breaks provided at home. Coordinated multidisciplinary and multiagency support should be readily available, with clear information provision and easy routes of access [47].

Traditional Chinese medicine covered by Taiwan’s NHI includes powdered Chinese herbal preparations, acupuncture, electropuncture, moxibustion, and manipulative therapy, health and nutrition education, and is highly used among children and young adults with life-limiting conditions. It has been previously found that children and adolescents with cancer, who suffered from drowsiness, lack of energy, and pain were more likely to use acupuncture [48]. Although there is good evidence in the literature that traditional Chinese medicine helps reduce cancer-related fatigue and chemotherapy-induced nausea and vomiting, and lowering infection rates in patients with leukemia [49], there is conflicting evidence regarding the treatment for dyspnea [50]. More work is needed to understand the effectiveness of traditional Chinese medicine in palliative care.

Previous study estimates 42% parents reported making use of psychological support services [51], which usually included self-support groups, psychological, and spiritual counselling [52]. Our findings expand the knowledge in the pediatric literature that there is a growing trend in the patient use of psychotherapy. Mental health professionals should be prepared to care for a growing number of children and young adults with life-limiting conditions with basic knowledge of palliative care. They should also be included within pediatric palliative care networks to ensure good co-ordination of care.

### Strength and limitations of the work

This is the first population-based study investigating specialized palliative care utilization in children and young adults with a life-limiting condition. The data sources were from national datasets; therefore, there was a strong backup for uploaded demographic information and reliable service use information. This study differs from a previous one [1] in that it only includes individuals who have had an inpatient or outpatient service recorded for a life-limiting condition in the current year. This ensures that the individuals being considered are still in need of medical care and are currently experiencing life-limiting conditions. While this may result in a decrease in estimated prevalence due to patients lost to follow-up [30], it ultimately increases accuracy.

This study has limitations. Firstly, there may have been lost cases from those who survived after a perinatal life-limiting condition and did not have a record of life-limiting condition diagnosis after their NHI enrollment procedures were completed (which should be done within 60 days of birth). However, this also means that we may have less numbers of infants who had a life-threatening event around the time of birth but were no longer considered as having a life-limiting condition in the year of birth, compared with the previous study [1].

Secondly, about 10% of life-limiting condition ICD-10-CM codes did not have a corresponding ICD-9-CM code; therefore, we may have underestimated the prevalence of life-limiting conditions between 2008 and 2015. Thirdly, a child or young adult was only required to have one recording of a life-limiting condition in any clinic or hospital to be included in these analyses. Children and young adults diagnosed with cancer may remain this record even if they had achieved full remission and were no longer life-limiting. Therefore, we may be capturing some cancer survivors who may not require palliative care. If individuals were not admitted to a clinic or hospital in a particular year, they were excluded from the prevalence estimation. As a result, individuals with poor compliance to medical follow-ups may be missed. Moreover, the diagnosis’s staging and the disease’s severity were unknown due to data unavailability. It is unclear whether individuals in each diagnostic group with specialized palliative care received timely access. Finally, this study did not include those with uninsured healthcare services, e.g., pastoral care and bereavement services, which were highly been used in parents of neonates [51].

## Conclusions

The prevalence of children and young adults with a life-limiting condition has increased markedly over this ten-year study period, with the highest prevalence in those aged 21–25 years. The use of specialized palliative care and other healthcare services has also increased, except for traditional Chinese medicine. Specialized palliative care should expand service populations to ensure that service access is available to all life-limiting diagnostic groups. All healthcare sectors should put greater focus on the needs of this population, particularly those highly requiring such services or those having increased such usage.

## Data Availability

The datasets used and/or analyzed during the current study are available from the corresponding author on reasonable request.

## Abbreviations

CI: Confidence interval
ICD: Classification of Diseases
NHI: National Health Insurance
